# Tumor-to-stroma cd8^+^ t cells ratio combined with cancer-associated fibroblasts: an innovative approach to predicting lymph node metastases of cervical cancer

**DOI:** 10.1007/s00432-023-05578-1

**Published:** 2024-02-19

**Authors:** Shuangshuang Guo, Peiyu Chen, Yang Yang, Wenfei Wei, YuHua Pan, Fanke Zeng, Liangsheng Fan, Wei Wang

**Affiliations:** 1 Department of Obstetrics and Gynecology, Guangzhou Medical University, The First Affiliated Hospital of Guangzhou Medical University, Guangzhou, 510120 Guangdong China; 2 The Six Affiliated Hospital, Guangzhou Medical University, Qingyuan, 511518 Guangdong China; 3 Department of Gynecology, Zhuhai People’s Hospital (Zhuhai Hospital Affiliated With Jinan University), Zhuhai, Guangdong China

**Keywords:** Cervical cancer, Cancer-associated fibroblasts, Lymph node metastases, Prediction model, Tumor-to-Stroma CD8^+^ T cells ratio

## Abstract

**Purpose:**

Precise identification of lymph node metastases is vital for the management of cervical cancer. However, the existing diagnostic methods for lymph node metastases have certain drawbacks. In this study, we aim to explore the expression of cancer-associated fibroblasts (CAFs) and tumor-to-stroma CD8^+^ T cells ratio (CD8^+^ T cells T:S ratio) and its association with lymph node metastases of cervical cancer.

**Methods:**

Hundred and ten cervical cancer tissues and 39 biopsy tissues from patients were investigated immunocytochemically for the expression of CAFs and CD8^+^ T cells. The statistical correlation analysis was carried out using the SPSS system.

**Results:**

A strong and statistically significant negative correlation (*r*= − 0.690; *P* < 0.001) was observed between CAF density and CD8^+^ T cells T:S ratio. Not only were CAFs density and CD8^+^ T cells T:S ratio correlated with lymph node metastases respectively (*P* < 0.001), but the combination of them also significantly correlated with lymph node metastases (*P* < 0.001). Then, we constructed the combined diagnosis model (Logit (*P*) = − 4.446 + 0.300 × CAFs + 0.752 × CD8+ T cells T:S Ratio) of cervical cancer lymph node metastases. ROC curves analysis showed that the ROC curves areas for CAFs, CD8^+^ T cells T:S ratio, and a combination of both are 0.879, 0.747, and 0.951. Then, the prediction model was verified by biopsy specimens and consistent results were obtained.

**Conclusions:**

The combination of CAF density and CD8^+^ T cells T:S ratio has a significant predictive value for lymph node metastases in patients with cervical cancer.

**Supplementary Information:**

The online version contains supplementary material available at 10.1007/s00432-023-05578-1.

## Introduction

Tumor invasion and lymph node metastases are the main causes of death in cervical cancer patients (Sundström and Elfström 2020; Sung et al. 2021). Currently, the standard treatment for early cervical cancer is primary surgical intervention, which involves radical hysterectomy and pelvic lymph node dissection (PLND) (Nitecki et al. 2020). Previous studies have reported a notable correlation between the number of excised lymph nodes and the disease-free survival rates among node-positive patients with cervical cancer (Pieterse et al. 2007). This suggests that adequate removal of lymph nodes in cervical cancer patients with positive lymph nodes appears to be a critical factor for optimal treatment outcomes. However, there is no definitive evidence indicating that extensive lymphadenectomy in negative lymph nodes of cervical cancer confers a survival advantage. Additionally, lymphadenectomy may develop intraoperative complications including hemorrhage, lymphocyst, and lymphedema (Mehra et al. 2010). More importantly, lymph nodes, especially tumor-draining lymph nodes (TDLNs), have been recognized as an essential component in cancer immunology and anti-cancer immunotherapies (Prokhnevska et al. 2023). In consideration of operative complications and lymph nodes’ clinical potential in anti-cancer immunity, precise identification of lymph node metastases is vital for the management of cervical cancer.

At present, lymph node status can be assessed pre-operatively with MRI, CT, and PET-CT. The mean accuracy of lymph node metastase detection was 86% (MRI) and 81% (CT) respectively(Boss et al. 2000). PET-CT examination only improves the accuracy of lymph node metastases with lesions > 10 mm (Lee and Atri 2018). Sentinel lymph node (SLN) biopsy is a more targeted method of assessing the spread of apparent early‐stage cervical cancer (Mathevet et al. 2021). However, the false negative rate of SLN biopsy can reach 16.6% due to the limitations of tracing methods and pathological detection techniques (Nagar, et al. 2021). Therefore, we can explore other approaches to improve the detection rate of lymph node metastasis.

Considerating the relationship between TDLN and tumors, we raise a question: whether lymph node metastases can be predicted through tumor-infiltrating immune cells? In 2006, a paper published in *Science* proposed that evaluating the tumor-infiltrating immune cells is a more accurate indicator of patient survival compared to traditional histopathological techniques (Galon et al. 2006). Subsequently, CD8^+^ T cells and CD57^+^ NK cells have been shown to provide prognostic values in several human malignancies (Chirica et al. 2015; Lee et al. 2006; Fang et al. 2017). Among these tumor-infiltrating immune cells, CD8^+^ T cells are essential in the immune system's resistance to tumor immune response and can directly eliminate tumor cells through their cytotoxic capabilities (Dong 2021; DeBerardinis 2020). Additionally, a variety of stroma cells, such as cancer-associated fibroblasts (CAFs), have also received increasing attention recently (Xiao and Yu 2021). For instance, CAFs can participate in tumor immunosuppression by limiting T cells in the extracellular matrix or affecting the function of CD8^+^ T cells, thereby promoting tumor lymph node metastases (Mariathasan et al. 2018; Wei et al. 2017). Therefore, both tumor-infiltrating immune cells and stroma cells have the potential to predict tumor metastases.

In this study, the number of CAFs and tumor-to-stroma CD8^+^ T cells ratio (CD8^+^ T cells T:S ratio) in invasive cervical cancer were observed to explore their clinical significance and predictive value for lymph node metastases.

## Methods

### Patients and specimens

110 cases (2012–2019) of cervical cancer and 39 biopsy tissues (2014–2019) were obtained from the archived records of the First Affiliated Hospital of Guangzhou Medical University. These tissue samples were formalin-fixed and embedded in paraffin. The research protocol was approved by the relevant institutional review boards of the hospital. The original staging information of included patients was assessed according to the FIGO 2009. We re-staged the original staging information using the updated FIGO 2018 staging guidelines. For example, patients in stages I–II were reclassified as stage IIIC based on the presence of lymph node involvement. Detailed information regarding the demographic, clinical, and histological features of the participants can be found in Table S1 and Table S2.

### Immunohistochemistry

To prepare the tissue sections, the paraffin was eliminated by incubating them at a temperature of 60 °C for an hour, followed by three washes in xylene to ensure complete dewaxing. Subsequently, the sections underwent gradual rehydration using a series of alcohol and distilled water. To inhibit endogenous peroxidase activity, the slides were incubated for 30 min in a solution of 0.3% H_2_O_2_. Antigen retrieval was achieved by subjecting the sections to pressure-cooking in a citric acid buffer. Then, the sections were incubated overnight with primary monoclonal antibodies, such as anti-α-SMA and anti-CD8 + . To visualize the reaction products, a streptavidin–biotin immunoperoxidase complex was utilized as a secondary antibody complex, with diaminobenzidine (DAB) as the chromogen. Hematoxylin was used for counterstaining, and the sections were mounted with a permanent mountant called DPX.

### Image analysis and counting

**Fig. 1:**
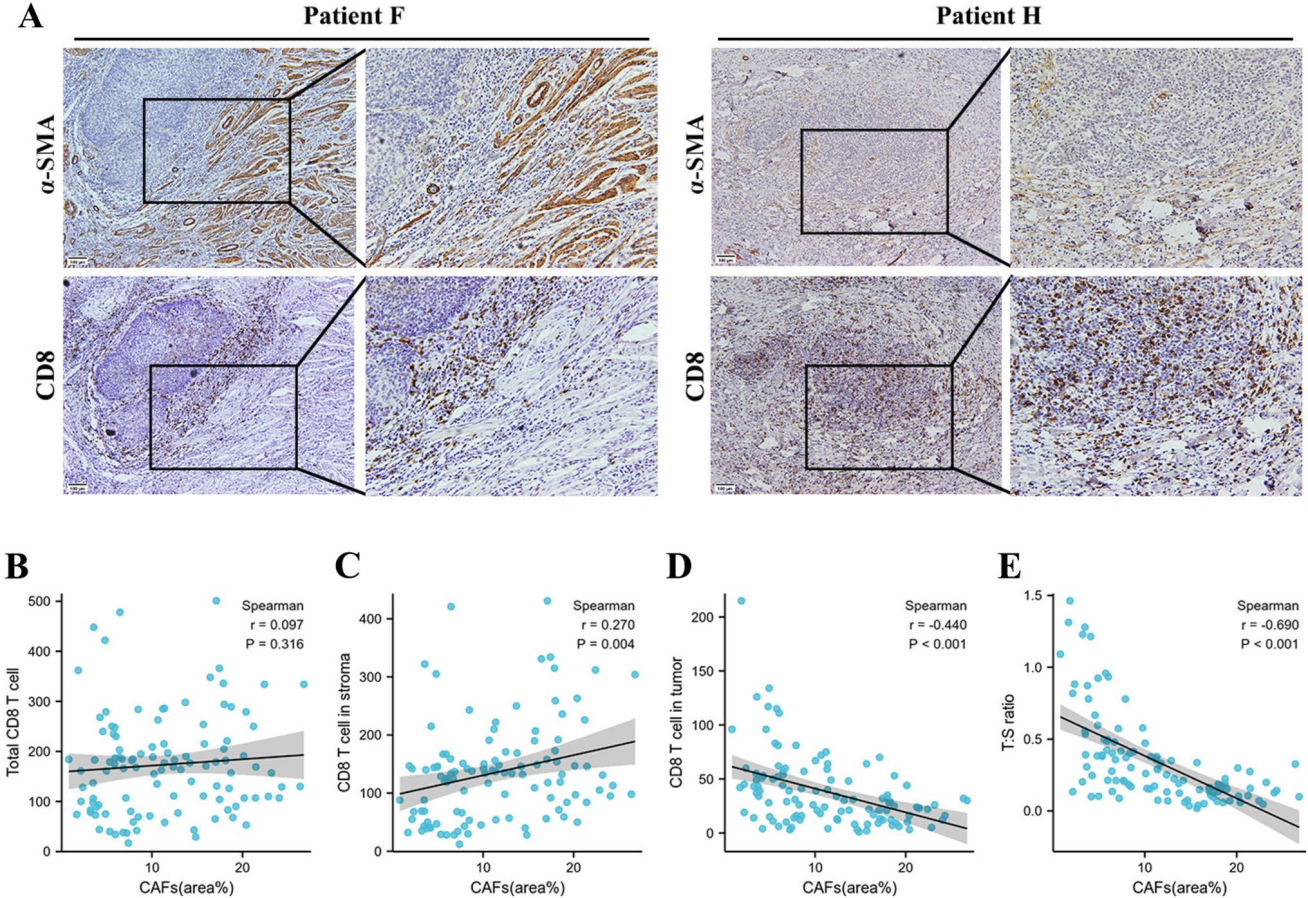
CAF density is correlated with CD8^+^ T cell density and location in cervical cancer. **A** The CAF distribution and CD8^+^ T cells infiltration degree in cervical cancer showed by immunohistochemical staining. CAFs distribution areas and the number of total CD8 + T cells (**B**), CD8 + T cells in stroma (**C**) or tumor (**D**), and CD8 + T cells T:S ratio (**E**) were analyzed in the same 110 study population respectively, statistical by Spearman’s correlation test

All image processing was performed using Image J software. To avoid overestimating the total area, any large void regions in the images were manually excluded. For bright field images, individual raw files were subjected to color deconvolution, using either HDAB or α-SMA. The appropriate color channel was then thresholded and converted into a binary image, allowing for the calculation of the percentage of positive area relative to the total sample area. The average area fraction was obtained by analyzing multiple images per patient (*n* = 3), yielding the final patient percentage as an indicator of CAF expression (Alcaraz et al. 2019). For the count of CD8^+^ T cells, the presence of brown particles on the lymphocyte membrane or cytoplasm was regarded as positive staining, and three areas (hot spots) with the most abundant positive staining lymphocytes were selected under high magnification (20X) field of vision to take micrographs. The intratumor and stromal positive cells were counted and their ratios were calculated. Among them, those greater than or equal to the median had a high inter-cancer ratio, and those less than the median had a low inter-cancer ratio. (Fig. 1A).

### Statistical analysis

Statistical analysis was performed using SPSS version 25.0. The Spearman correlation test was used to determine the correlation between expressions of CAFs and CD8^+^ T cells. The association of CAFs and CD8^+^ T cells T:S ratio and the combination of the two with clinical characteristics were analyzed by the *χ*^2^ test. Multiple logistic regression analysis was used to analyze the value of the combination of the CAFs and the CD8^+^ T cells T:S ratio in predicting the lymph node metastases of cervical cancer. The Chi-square test was used to verify the coincidence rate of the prediction model on biopsy specimens. All *P* values < 0.05 were considered statistically significant.

## Results

### CAF density is correlated with CD8^+^ T cell density and location in cervical cancer

To investigate the density and location of CD8^+^ T cells and CAFs in cervical cancer, we analyzed the expression of α-SMA and CD8 by immunohistochemical (IHC) staining in 110 cervical cancer specimens individually. The clinicopathological characteristics are shown in Table S1. IHC staining results showed that the CAFs were mainly distributed in the TME around the tumor, and the CD8^+^ T cells were located in both the tumor and stroma (Fig. 1A).

Given the proposed relationship between CD8 + T cells and CAFs in tumors, we carried out a correlation analysis between the CAFs and CD8^+^ T cell density using Spearman’s correlation test. As shown in Fig. 1B, there is no correlation (*r* = − 0.097; *P* = 0.316) between the CAF density and the number of total CD8^+^ T cells. As previous studies reported that CAFs can participate in tumor immunosuppression by limiting T cells in the extracellular matrix, we were interested in whether CAFs can determine the distribution of CD8^+^ T cells in tumors and stroma. To test this, we then analyzed the correlation between the CAF density with CD8^+^ T cells in tumors and CD8^+^ T cells in stroma individually. After integrating data from 110 cases, the CAF density has a negative correlation (*r* = − 0.440; *P* < 0.001) with CD8^+^ T cells in the tumor and a positive correlation (*r* = − 0.270; *P* = 0.004) with CD8^+^ T cells in the stroma (Fig. 1C and D). Subsequently, a novel parameter referred to as CD8^+^ T cells T:S ratio was employed. Statistical correlation analysis also revealed a highly significant negative correlation (*r* = − 0.690; *P* < 0.001) between CAF density and CD8^+^ T cells T:S ratio in cervical cancer. One possible explanation for these data is that CAFs may suppress CD8^+^ T-cell infiltration in tumors.

### CAF density and CD8^+^ T cells T:S ratio are significantly correlated with lymph node metastases

**Fig. 2:**
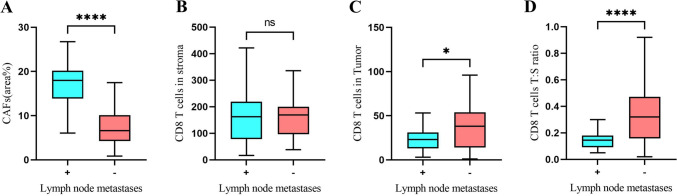
CAF density and CD8^+^ T cells T:S ratio are significantly correlated with lymph node metastases in cervical cancer. Correlation analysis between lymph node metastases and CAFs distribution **A**, CD8^+^ T cells in stroma **B**, CD8^+^ T cells in tumor **C**, and CD8^+^ T cells T:S ratio **D**

We hypothesized that the number and location of CAFs and CD8 + T cells might determine the prognosis of patients with cervical cancer. Based on the expression pattern of α-SMA, these patients were divided into a CAF^high^ group (upper half) and a CAF^low^ group (lower half). Other factors were also classified into high and low groups according to the same criteria (upper half into the high group and lower half into the low group) (Table S3). The relationship between CAF density or the number of CD8^+^ T cells and clinical characteristics of cervical cancer are summarized in Table S3. The distribution of CAFs had a significant correlation with lymph node metastases (*P* < 0.001) and tumor stage (*P* < 0.001) but did not correlate with other clinicopathological parameters. Additionally, the CD8^+^ T cells T:S ratio was also correlated with lymph node metastases (*P* < 0.001) and tumor stage (*P* < 0.001). Both the CD8^+^ T cells in tumors and stroma did not relate to other clinicopathological parameters (Table S3). Further, we focused on lymph node metastases and found that the CAF density (*P* < 0.001) and CD8^+^ T cells T:S ratio (*P* < 0.001) had a significant correlation with lymph node metastases (Fig. 2).

We further classified these patients into four different groups (CAFs^high^ T:S ratio^high^, CAFs^high^ T:S ratio^low^, CAFs^low^ T:S ratio^high^, and CAFs^low^ T:S ratio^low^) according to CAFs density and CD8^+^ T cells T:S ratio in cervical cancer. We found that the combination of CAF density and CD8^+^ T cells T:S ratio had a significant correlation with lymph node metastases (*P* < 0.001) and tumor stage (*P* < 0.001), but did not correlate with other clinicopathological parameters (Table 1).

**Table 1:** Relationship between the combination of CAFs and CD8 T:S ratio and clinicopathological features

	CAFs^high^ T:S ratio^high^	CAFs^high^ T:S ratio^low^	CAFs^low^ T:S ratio^high^	CAFs^low^ T:S ratio^low^	*χ*^2^	*Ρ*
Age						
< 50	6	18	15	5	0.402	0.940
≥ 50	8	24	26	8		
Lymph node metastases						
Present	8	30	2	1	46.598	< 0.001^*^
Absent	6	12	39	12		
Tumor stage						
IA	1	0	0	0	67.571	< 0.001^*^
IB	1	3	29	7		
IIA	2	5	7	4		
IIB	2	4	3	0		
IIIC	8	30	2	1		
Histological type						
Adenocarcinoma	3	6	2	1	3.783	0.286
Squamous cell carcinoma	11	36	39	12		
Differentiation						
G1	2	1	0	1	6.763	0.343
G2	9	33	31	9		
G3	3	8	10	3		
Perineural invasion						
Present	0	3	2	1	1.923	0.589
Absent	14	39	39	12		
Venous invasion						
Present	5	11	7	2	0.719	0.438
Absent	9	31	34	11		

Bold coefficients and asterisks indicate *P* < 0.001

### Better predictive performance was achieved for lymph node metastases in the combination of CAF density and CD8^+^ T cells T:S ratio

**Fig. 3:**
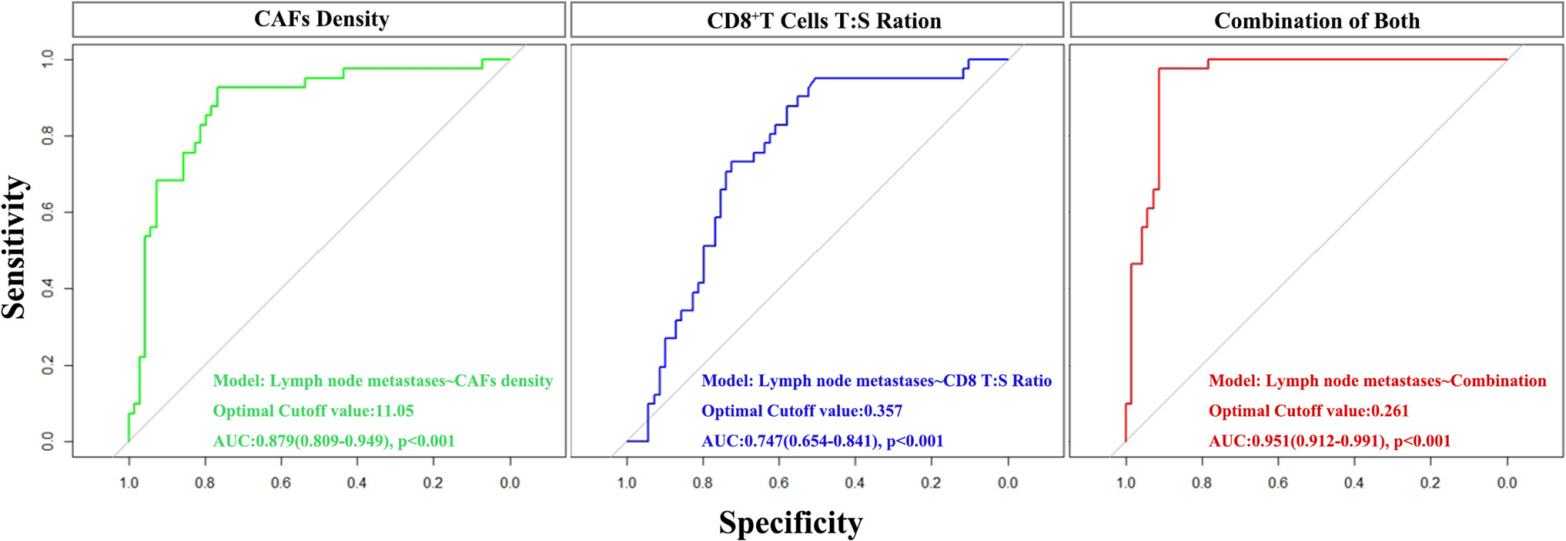
The predictive accuracy of CAF density, CD8^+^ T cells T:S ratio, and a combination of both for lymph node metastasis were compared by the AUC of the receiver operating characteristic (ROC) curve

Based on the significant correlations between lymph node metastases with CAFs and CD8 + T cells, we wanted to know if CAFs and CD8 + T cells have the potential to predict tumor metastases. First, we used the logistic regression analysis to construct the combined diagnosis model of cervical cancer lymph node metastases. The logistic regression model was established with lymph node metastases as the dependent variable and CAFs density and CD8^+^ T cells T:S ratio as the independent variables. The resulting model is shown as Logit (P) = –4.446 + 0.300 × CAFs + 0.752 × CD8^+^ T cells T:S Ratio. The likelihood ratio test showed that the difference in the regression model was statistically significant (*χ*^2^ = 51.814, *P* < 0.001). Also, the predictive accuracy of CAFs and CD8^+^ T cells T:S ratio was compared by the AUC of the receiver operating characteristic (ROC) curve (Fig. 3). ROC curves analysis showed that the ROC curves areas for CAFs, CD8^+^ T cells T:S ratio, and combination of both are 0.879 (95%CI 0.809–0.946), 0.747 (95%CI 0.654–0.841) and 0.951 (95%CI 0.912–0.991), respectively, indicating that the area under the curve was the largest in the combined detection of the two indicators (Fig. 3 and Table S4). It revealed that the predictive accuracy of the combined detection is higher than the individual indicators of lymph node metastases.

### Verification of the validity of the prediction model by biopsy specimens

**Fig. 4:**
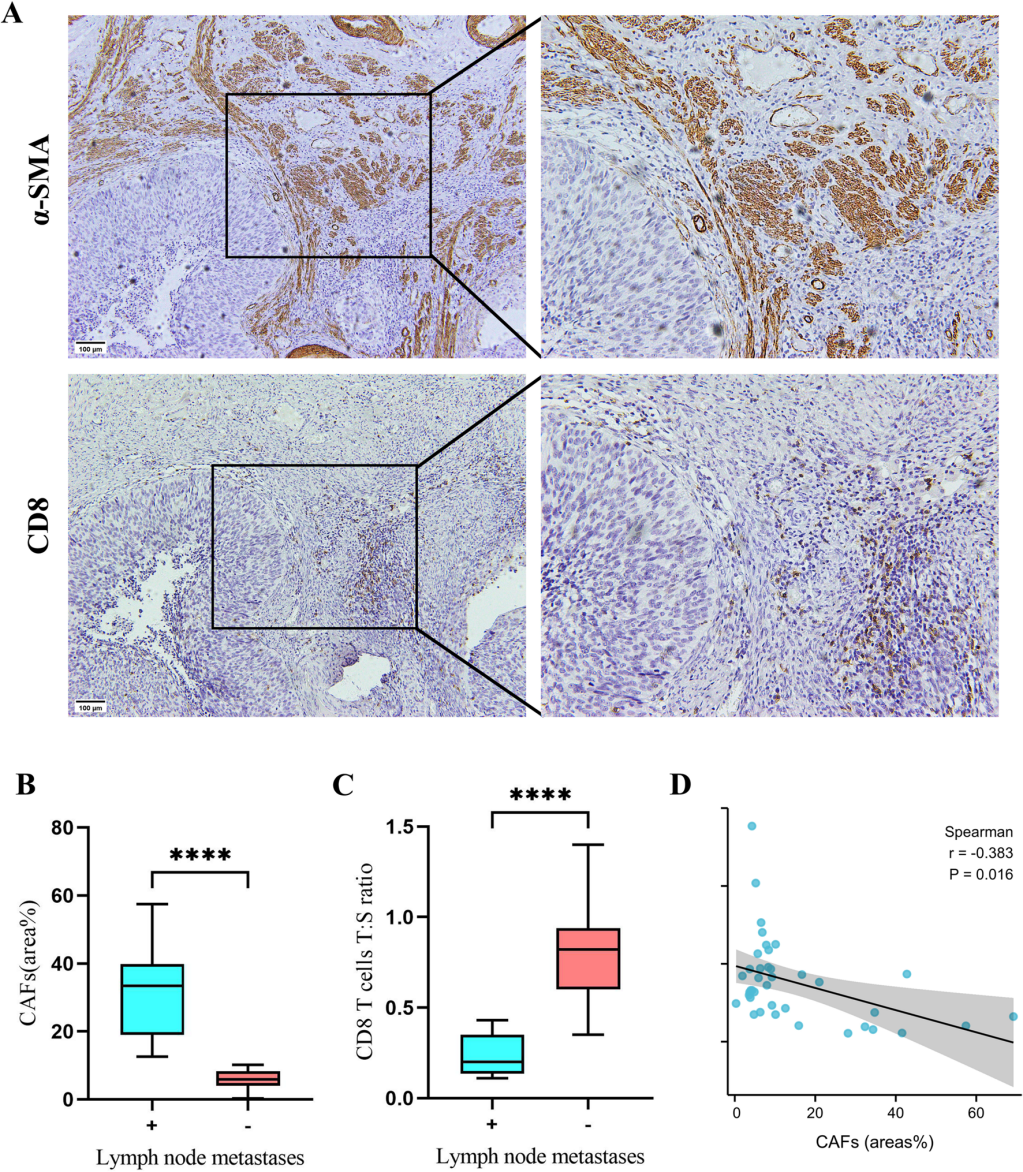
Evaluate the discriminating ability of lymph node metastasis of the prediction model by biopsy specimens from 39 patients

To evaluate the discriminating ability of lymph node metastases of the prediction model, we collected the biopsy specimens from 39 patients (Table S2). The results, like before, showed that CAF expressions and CD8^+^ T cells T:S ratio correlate (*r* = − 0.383; *P* = 0.016), and all were associated with lymph node metastases (*P* < 0.001) (Fig. 4). Furthermore, according to the indicators’ optimal threshold of the prediction model, we analyzed the coincidence rate of lymph node metastases between prediction and postoperative pathological diagnosis in biopsy specimens. The validity of the three indicators was found to be desirable, especially the combined detection (Table S5).

## Discussion

In this study, we found that the CAF density has a relationship with the CD8^+^ T cells in the tumor, stroma, and T:S ratio in cervical cancer. We also found that the combination of CAF density and CD8^+^ T cells T:S ratio had a significant correlation with lymph node metastases and tumor stage. Additionally, we constructed the combined diagnosis model of lymph node metastases. Then, the prediction model was verified by biopsy specimens and consistent results were obtained.

Previous studies demonstrated that CAFs can promote tumor development, invasion, and metastases (Yamauchi et al. 2020; Denton et al. 2018). Additionally, CAFs also shape the extracellular matrix to form an osmotic barrier to prevent the deep penetration of immune cells, such as CD8 + T cells, into tumor tissues, thereby reducing the tumor-killing effect (Chen and Song 2019). When CD8 + T cells are arrested within the stromal area, their activities are inhibited and allow the tumor to progressively evade the surveillance and assault of CD8 + T cells, ultimately leading to immune evasion and the development of tumor metastases. On the one hand, the collagen matrix produced by CAFs may form a physical barrier (Grasset et al. 2018), which mediates the exclusion of T cells. The alignment, spacing, and density of collagen fibers within the tumor microenvironment regulate the localization and movement of CD8 + T cells within the tumor stroma. The enhanced deposition of collagen matrix hampers the capacity of CD8 + T cells to establish contact with cancer cells (Modugno et al. 2019). On the other hand, CAFs affected CD8^+^ T cell function by secreting cytokines and regulating antigen presentation (Goehrig et al. 2019; Lakins et al. 2018). With the presence of CAFs, proliferating T cells produced less IFN-γ, TNF-α, and CD107a. This suggested that the presence of CAFs promoted the upregulation of co-inhibitory receptors on CD8 + T cells, resulting in suppressed immune function (Gorchs et al. 2019). These studies validate the feasibility of utilizing the combination of CAF and CD8 in predicting lymph node metastasis. However, the retrospective design, the modest number of patients, and the single-center analysis are the main weaknesses. Subsequently, we will conduct a multicenter and multicenter prospective study.

Lymph node metastasis is an independent predictor of surgical treatment and prognosis of cervical cancer (Cohen et al. 2019). NCCN guidelines recommend extensive hysterectomy with systemic pelvic lymphadenectomy as the standard procedure for early cervical cancer (Koh et al. 2019). However, studies have shown that more than 80% of early cervical cancer patients suffer from adverse reactions caused by the excision of normal lymph node tissue (Raspagliesi and Bogani 2019). Enhancing the preoperative detection rate of lymph node metastases in cervical cancer is a challenging and crucial aspect of its clinical management. In this investigation, we assess the relationship between the CAF density and the T:S ratio of CD8 + T cells, aiming to determine their correlation and predictive value in diagnosing lymph node metastases. The ultimate goal is to minimize unnecessary "lymphadenectomy" procedures and enhance clinical prognosis through a more accurate and personalized treatment approach. The establishment of this predictive model by the ROC curve is capable of guiding the surgical methods well and further reducing the complications after lymphadenectomy. Furthermore, it also has the potential to evaluate the effectiveness of immunotherapy for advanced cervical cancer.

## Conclusion

**Fig. 5:**
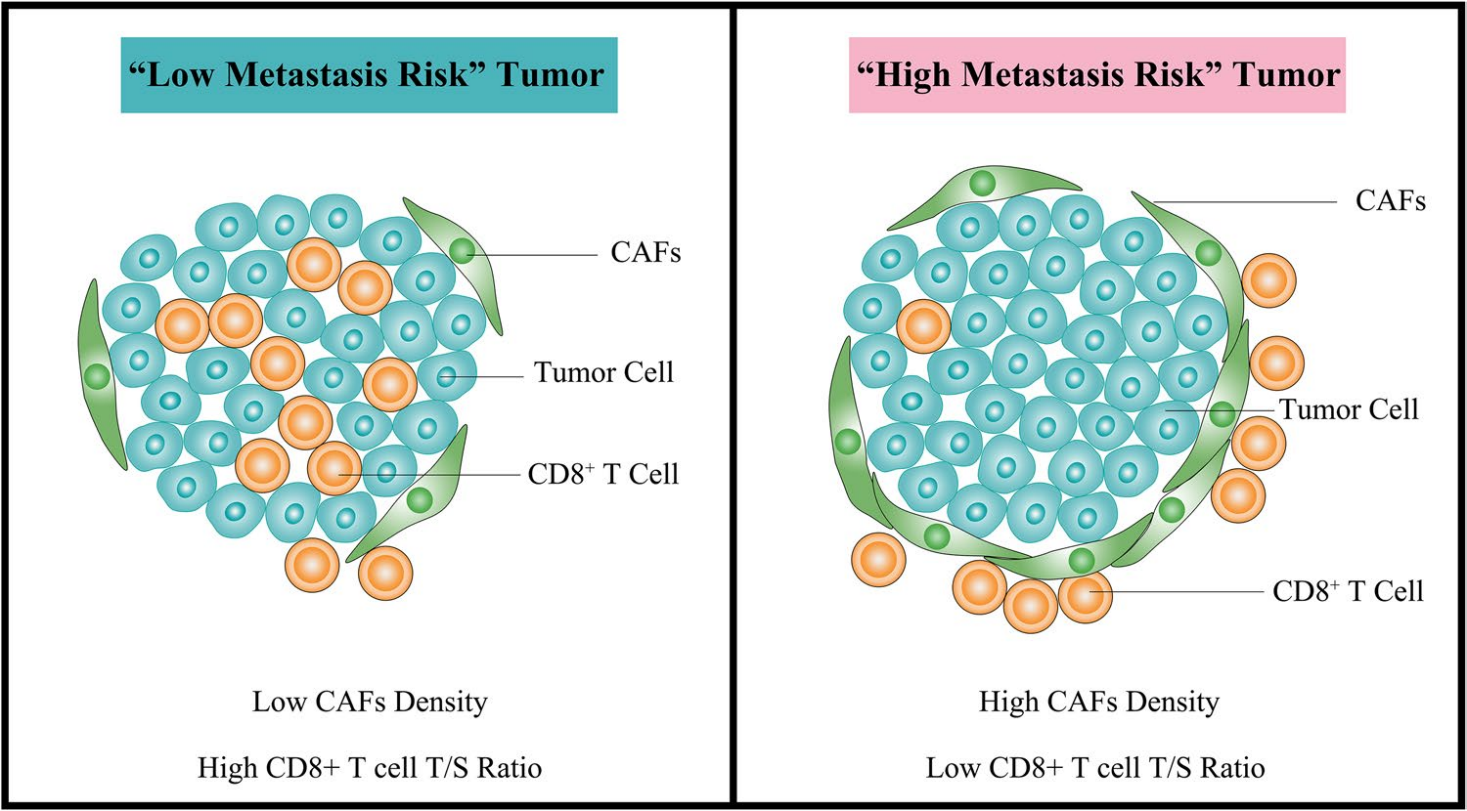
Illustrative model showing the relationship between lymph node metastasis and CAF density, as well as CD8^+^ T cells T:S ratio

This study confirms that CAF density has a negative correlation with CD8^+^ T cells T:S ratio in cervical cancer. The combination of CAF density and CD8^+^ T cells T:S ratio has a significant predictive value for lymph node metastases in patients with cervical cancer (Fig. 5).

### Supplementary Information

Below is the link to the electronic supplementary material.

Supplementary file1 (DOCX 17 KB)

Supplementary file2 (DOCX 17 KB)

Supplementary file3 (DOCX 23 KB)

Supplementary file4 (DOCX 15 KB)

Supplementary file5 (DOCX 16 KB)

## Data Availability

The datasets generated during and/or analyzed during the current study are available from the corresponding author upon reasonable request.
