# Supplementary material for: Tumor-to-stroma cd8+ t cells ratio combined with cancer-associated fibroblasts: an innovative approach to predicting lymph node metastases of cervical cancer
Source: J Cancer Res Clin Oncol. 2024 Feb 19;150(2):93. doi: 10.1007/s00432-023-05578-1 (PMC10874907; doi:10.1007/s00432-023-05578-1)
Supplement: Supplementary file 1 — Supplementary file1 (DOCX 17 KB) [file 432_2023_5578_MOESM1_ESM.docx]

**Table S1** Characteristics of cervical cancer study population (n=110).

| Variable | Number | Percent % |
| --- | --- | --- |
| Age | | |
| <50 | 44 | 40.00 |
| ≥50 | 66 | 60.00 |
| Lymph node metastases | | |
| Present | 41 | 37.27 |
| Absent | 69 | 62.73 |
| Tumor Stage (FIGO 2018) | | |
| IA | 1 | 0.91 |
| IB | 40 | 36.36 |
| IIA | 19 | 17.27 |
| IIB | 9 | 8.18 |
| IIIC | 41 | 37.27 |
| Histological type  Adenocarcinoma  Squamous cell carcinoma  Differentiation  G1  G2  G3  Perineural invasion  Present  Absent  Venous invasion  Present  Absent | 12  98  4  82  24  6  104  25  85 | 10.91  89.09  3.64  74.54  21.82  5.45  94.55  22.73  77.27 |
