# Supplementary material for: Tumor-to-stroma cd8+ t cells ratio combined with cancer-associated fibroblasts: an innovative approach to predicting lymph node metastases of cervical cancer
Source: J Cancer Res Clin Oncol. 2024 Feb 19;150(2):93. doi: 10.1007/s00432-023-05578-1 (PMC10874907; doi:10.1007/s00432-023-05578-1)
Supplement: Supplementary file 2 — Supplementary file2 (DOCX 17 KB) [file 432_2023_5578_MOESM2_ESM.docx]

**Table S2** Characteristics of biopsy study population (n=39).

| Variable | Number | Percent % |
| --- | --- | --- |
| Age  <50  ≥50 | 17  22 | 43.59  56.41 |
| Lymph node metastases  Present  Absent | 9  30 | 23.08  76.92 |
| Tumor Stage  IA  IB  IIA  IIB  IIIC  Differentiation  G1  G2  G3  Venous invasion  Present  Absent | 0  21  9  0  9  4  26  9  13  26 | 0.00  53.85  23.07  0.00  23.07  10.25  66.67  23.08  33.33  66.67 |
