# Supplementary material for: Tumor-to-stroma cd8+ t cells ratio combined with cancer-associated fibroblasts: an innovative approach to predicting lymph node metastases of cervical cancer
Source: J Cancer Res Clin Oncol. 2024 Feb 19;150(2):93. doi: 10.1007/s00432-023-05578-1 (PMC10874907; doi:10.1007/s00432-023-05578-1)
Supplement: Supplementary file 3 — Supplementary file3 (DOCX 23 KB) [file 432_2023_5578_MOESM3_ESM.docx]

**Table S3** Clinicopathological parameters of patients with CAFs and CD8^+^T cells.

|  | CAFs | | χ^2^ | Ρ | CD8^+^T cell in stroma | | χ^2^ | Ρ | CD8^+^T cell in tumor | | | χ^2^ | | Ρ | | T:S ratio | | | | χ^2^ | | Ρ |
| --- | --- | --- | --- | --- | --- | --- | --- | --- | --- | --- | --- | --- | --- | --- | --- | --- | --- | --- | --- | --- | --- | --- |
|  | High | Low |  |  | High | Low |  | | High | Low | |  | | High | | | Low | |  | | |  |
| Age  <50  ≥50  Lymph node metastases  Present  Absent  Tumor Stage  IA  IB  IIA  IIB  IIIC  Histological type  Adenocarcinoma  Squamous cell carcinoma  Differentiation  G1  G2  G3  Perineural invasion  Present  Absent  Venous invasion  Present  Absent | 24  32  38  18  1  4  7  6  38  9  47  3  42  11  3  53  16  45 | 20  34  3  51  0  36  12  3  3  3  51  1  40  13  3  51  9  40 | 0.388  45.639  58.784  3.128  1.226  0.002  0.956 | 0.533  **＜0.001***  **＜0.001***  0.077  0.542  0.963  0.328 | 22  34  24  32  1  20  9  2  24  7  49  3  43  10  5  55  12  44 | 22  32  17  37  0  20  10  7  17  5  49  1  39  14  1  49  13  41 | 0.024  1.522  4.991  0.297  1.875  2.347  0.110 | 0.876  0.217  0.288  0.586  0.392  0.126  0.741 | 21  34  14  28  1  26  10  4  14  4  51  2  43  10  3  52  10  45 | 23  32  27  41  0  14  9  5  27  8  47  2  39  14  3  52  15  40 | 0.152  0.451  80886  1.497  0.865  0.000  1.297 | | 0.697  0.502  0.064  0.221  0.649  1.000  0.255 | | 21  34  10  45  1  30  9  5  10  5  50  2  40  13  2  53  12  43 | | | 23  32  31  24  0  10  10  4  31  7  48  2  42  11  4  51  13  42 | | 0.152  17.147  21.924  0.417  0.216  0.705  0.052 | 0.697  **＜0.001***  **＜0.001***  0.518  0.898  0.401  0.820 | |
