# Supplementary material for: Tumor-to-stroma cd8+ t cells ratio combined with cancer-associated fibroblasts: an innovative approach to predicting lymph node metastases of cervical cancer
Source: J Cancer Res Clin Oncol. 2024 Feb 19;150(2):93. doi: 10.1007/s00432-023-05578-1 (PMC10874907; doi:10.1007/s00432-023-05578-1)
Supplement: Supplementary file 4 — Supplementary file4 (DOCX 15 KB) [file 432_2023_5578_MOESM4_ESM.docx]

**Table S4** The predictive value of CAFs and CD8 T:S ratio in lymph node metastases of cervical cancer.

| Predictive indicator | Optimal threshold | Sensitivity (%) | Specificity (%) | AUC | 95% CI |
| --- | --- | --- | --- | --- | --- |
| CAFs | 10.861 | 0768 | 0.927 | 0.879 | 0.809-0.946 |
| T:S ratio | 0.365 | 0.507 | 0.951 | 0.747 | 0.654-0.841 |
| Combination of the two | 0.257 | 0.913 | 0.976 | 0.951 | 0.912-0.991 |
