# Supplementary material for: Tumor-to-stroma cd8+ t cells ratio combined with cancer-associated fibroblasts: an innovative approach to predicting lymph node metastases of cervical cancer
Source: J Cancer Res Clin Oncol. 2024 Feb 19;150(2):93. doi: 10.1007/s00432-023-05578-1 (PMC10874907; doi:10.1007/s00432-023-05578-1)
Supplement: Supplementary file 5 — Supplementary file5 (DOCX 16 KB) [file 432_2023_5578_MOESM5_ESM.docx]

**Table S5** The coincidence rate of lymph node metastases between prediction and postoperative pathological diagnosis in biopsy specimens according to the indicators.

| indicators | postoperative pathological diagnosis | n | prediction | | coincidence number | coincidence rate (%) | χ^2^ | P |
| --- | --- | --- | --- | --- | --- | --- | --- | --- |
|  |  |  | Yes | No |  |  |  |  |
|  | Yes | 9 | 8 | 1 | 8 | 88.89 | 28.547 | ＜0.001* |
| CAFs | No | 30 | 1 | 29 | 29 | 96.55 |  |  |
|  | Total | 39 | 9 | 30 | 37 | 94.87 |  |  |
|  | Yes | 9 | 7 | 2 | 7 | 77.78 | 19.721 | ＜0.001* |
| T:S ratio | No | 30 | 2 | 28 | 28 | 93.33 |  |  |
|  | Total | 39 | 9 | 30 | 35 | 89.74 |  |  |
|  | Yes | 9 | 9 | 0 | 9 | 100.00 | 33.930 | ＜0.001* |
| combination | No | 30 | 1 | 29 | 29 | 96.67 |  |  |
|  | Total | 39 | 10 | 29 | 38 | 97.44 |  |  |
